# Bilateral Bloody Pleural Effusions With Discordant Cellular Patterns: A Case of Concurrent Parapneumonic Effusion and Dressler Syndrome

**DOI:** 10.1002/rcr2.70764

**Published:** 2026-09-16

**Authors:** Yoshitsugu Narumi, Susumu Hoshino, Midori Yamakawa, Yoshiko Amano

**Affiliations:** ^1^ Department of Internal Medicine and Pulmonary Medicine Seikei‐kai Chiba Medical Center Chiba Japan

**Keywords:** Dressler syndrome, parapneumonic effusion, pleural effusion, pneumococcal pneumonia, *S. pneumoniae*

## Abstract

Bilateral pleural effusions typically suggest systemic aetiologies; however, concurrent distinct localised pathologies are rare. A 73‐year‐old man presented with fever and cough. Chest imaging revealed pneumonia and bilateral pleural effusions developed during antibiotic treatment. The right effusion was neutrophil‐dominant, consistent with parapneumonic effusion, while the left was lymphocyte‐dominant, suggesting Dressler syndrome triggered by pneumonia‐induced systemic inflammation. Both effusions were bloody, an appearance possibly exacerbated by the concomitant use of a direct oral anticoagulant and aspirin. The left effusion recurred months later but significantly improved following ibuprofen treatment, supporting an immune‐mediated aetiology. This case highlights that bilateral effusions can arise from different aetiologies and that pneumonia‐induced inflammation may trigger a latent immune response related to prior cardiac surgery.

## Graphical abstract

This case demonstrates a rare concurrent presentation of parapneumonic effusion and Dressler syndrome, which presented as bilateral bloody pleural effusions with discordant cellular profiles. The bloody nature was possibly exacerbated by the concomitant use of a direct oral anticoagulant and aspirin.

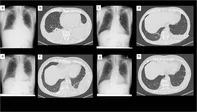

## 1 Introduction

The majority of pleural effusions result from congestive heart failure, pneumonia and cancer [1]. Generally, systemic conditions such as congestive heart failure cause bilateral effusions, whereas localised thoracic pathologies present as unilateral effusions. Parapneumonic effusions are a well‐recognised complication of bacterial pneumonia, particularly *Streptococcus pneumoniae* and are pathologically characterised by a neutrophil‐dominant exudate driven by acute inflammation [2]. Conversely, Dressler syndrome (post‐cardiac injury syndrome [PCIS]) is an immune‐mediated pleuropericarditis that classically presents within a few weeks to months following cardiac surgery, such as coronary artery bypass grafting (CABG), manifesting with lymphocyte‐dominant pleural fluid [3]. While both pneumonia and Dressler syndrome can independently cause pleural effusions, the concurrent presentation of these distinct, localised pathologies in a single patient—resulting in bilateral effusions with discordant cellular profiles—is exceedingly rare. Furthermore, it is known that the use of direct oral anticoagulants (DOACs) can induce spontaneous pleural bleeding [4]. In the present case, we hypothesise that the concomitant use of a DOAC and aspirin further complicated the underlying inflammatory effusions, leading to their bloody appearance. Bilateral pleural effusions arising from two distinct aetiologies are classically known as Contarini syndrome [5], and the present case represents one such combination. To our knowledge, a PubMed search identified no previous reports of a parapneumonic effusion and Dressler syndrome presenting concurrently.

Here, we report a highly unusual case of bilateral bloody pleural effusions where the right effusion was a parapneumonic exudate, and the left was suspected to be Dressler syndrome, potentially triggered by the pneumonia‐induced systemic inflammation. This case highlights the critical importance of evaluating bilateral effusions for independent aetiologies.

## 2 Case Report

On April 27, a 73‐year‐old man presented to the emergency department with a 1‐week history of fever and productive cough. His medical history included hypertension, diabetes mellitus and dyslipidaemia; a cerebral infarction resulting in left hemiparesis 15 years earlier; and CABG for acute coronary syndrome approximately 12 months prior to presentation. He had a 52‐pack‐year smoking history and had quit 1 year prior to presentation. There was no occupational dust exposure or history of trauma. His medications included antidiabetic agents and antithrombotic therapy with edoxaban and aspirin.

**FIGURE 1:**
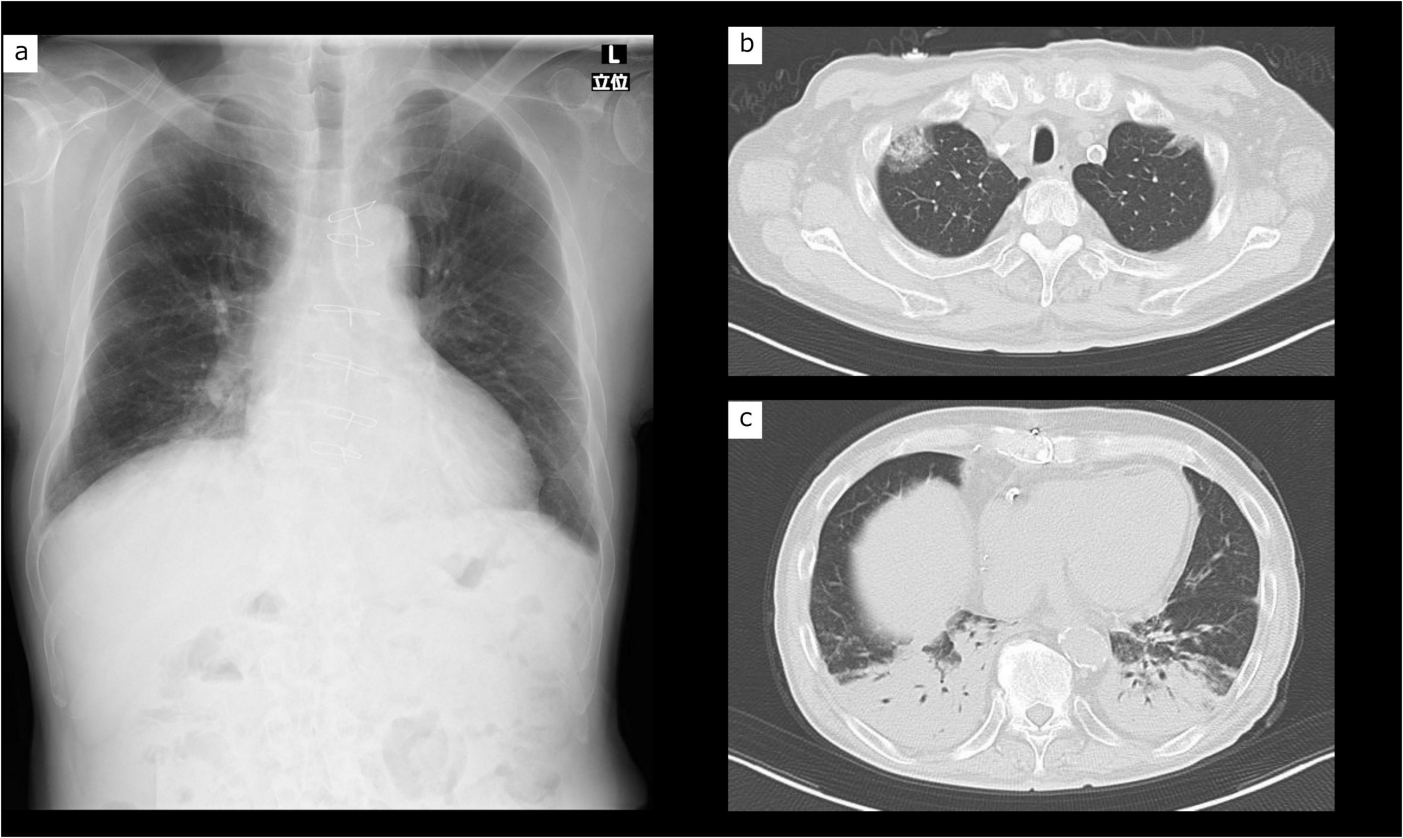
Imaging findings on admission. (a) Chest radiography and (b, c) computed tomography (CT) showing consolidations in the right upper lobe and bilateral lower lobes.

On admission, he was alert. Vital signs showed a temperature of 38.6°C, heart rate of 85 beats/min, blood pressure of 137/80 mmHg and oxygen saturation of 94% on 4 L/min via nasal cannula. Physical examination revealed decreased breath sounds at both lung bases. Chest radiography and computed tomography (CT) showed consolidations in the right upper lobe and bilateral lower lobes (Figure 1). Laboratory findings showed a marked neutrophil predominance and markedly elevated C‐reactive protein (CRP; Table 1). Arterial blood gas analysis on room air revealed severe hypoxaemia (partial pressure of oxygen [PaO_2_] 48.8 mmHg). Urinary pneumococcal antigen tested positive, and a concurrent sputum culture grew *S. pneumoniae* (2+). Together, these findings supported an active pneumococcal infection rather than a residual antigen from a past infection.

**TABLE 1:** Laboratory findings on admission.

Parameter	Value	Unit
Haematology		
White blood cells	6800	/μL
Neutrophil	86.5	%
Lymphocyte	7.5	%
Monocyte	4	%
Eosinophil	1	%
Basophil	1	%
Red blood cells	3.81	×10^6^/μL
Haemoglobin	11.1	g/dL
Haematocrit	33.8	%
Platelet	26.2	×10^4^/μL
Coagulation		
PT‐INR	1.46	
APTT	41.2	sec
**Biochemistry**		
Total protein	5.6	g/dL
Urea nitrogen	13.2	mg/dL
Creatinine	0.69	mg/dL
Total bilirubin	0.9	mg/dL
AST	25	IU/L
ALT	22	IU/L
LDH	208	IU/L
CK	45	IU/L
Glucose	220	mg/dL
HbA1c	8.1	%
BNP	192.5	pg/mL
Sodium	133	mEq/L
Potassium	3	mEq/L
Chloride	98	mEq/L
Serology		
C‐reactive protein	29.7	mg/dL
Arterial Blood Gas (room air)		
pH	7.431	
PaCO_2_	30.3	mmHg
PaO_2_	48.8	mmHg
HCO_3_–	19.8	mmol/L
Urinalysis		
Protein	±	
Glucose	4+	
Ketone bodies	2+	
Occult blood	2+	
Legionella antigen	Negative	
Pneumococcal antigen	Positive	
Microbiology		
Sputum culture	*S. pneumoniae* 2+	

Abbreviations: ALT, alanine aminotransferase; APTT, activated partial thromboplastin time; AST, aspartate aminotransferase; BNP, brain natriuretic peptide; CK, creatine kinase; HbA1c, glycated haemoglobin; HCO_3_^−^, bicarbonate; LDH, lactate dehydrogenase; PaCO_2_, partial pressure of carbon dioxide; PaO_2_, partial pressure of oxygen; PT‐INR, prothrombin time‐international normalised ratio.

**FIGURE 2:**
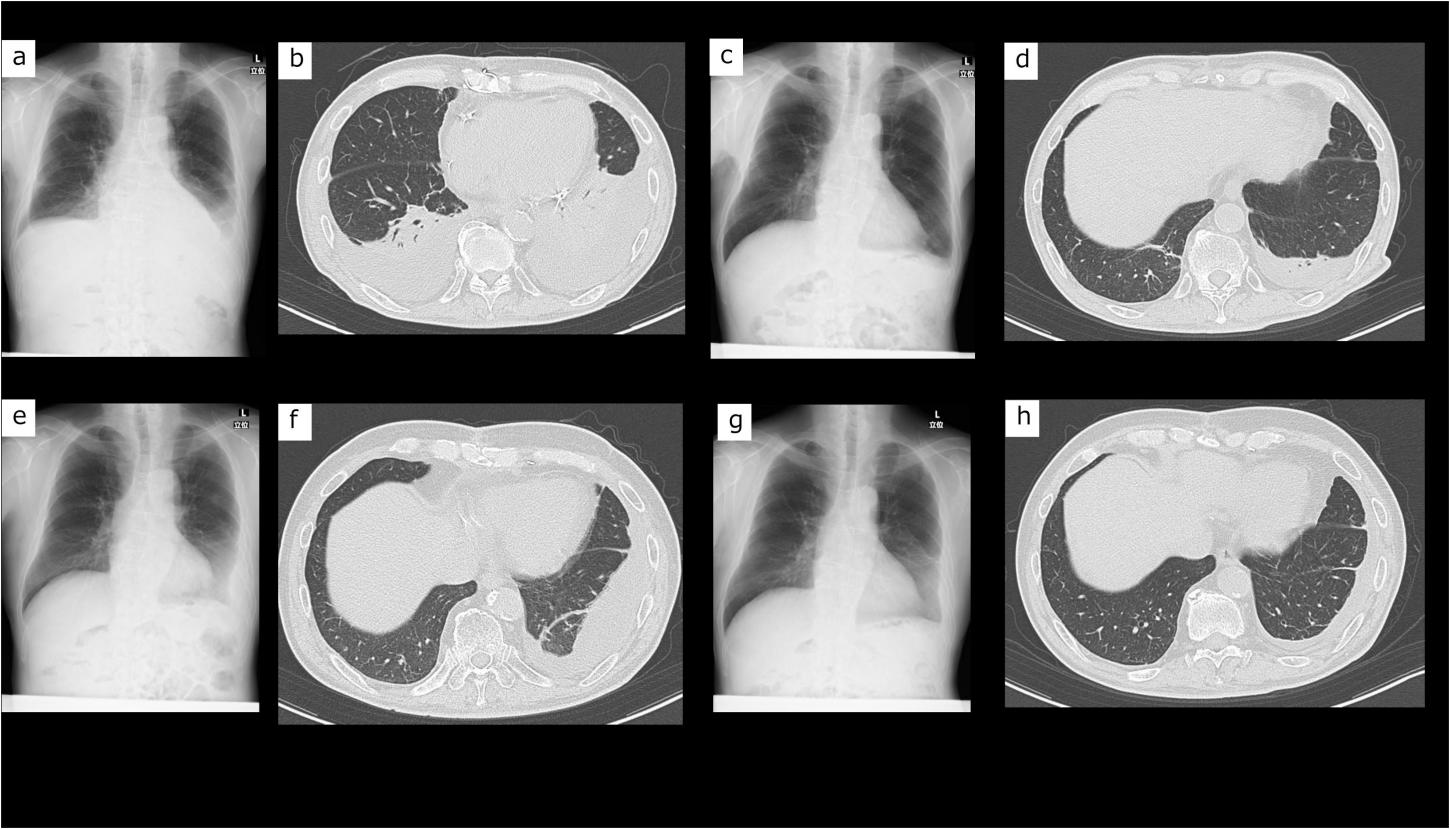
Clinical course of the pleural effusions. (a, b) Chest radiography and computed tomography (CT) on May 7 showing new‐onset bilateral pleural effusions. (c, d) Imaging on May 21 showing partial resolution of the pleural effusions. (e, f) Imaging on August 29 showing worsening of the left effusion. (g, h) Imaging on September 19 showing improvement after ibuprofen treatment.

Due to severe hypoxaemia, empiric therapy with piperacillin/tazobactam (13.5 g/day) was initiated. By hospital day 11 (May 7), chest radiography and CT showed resolution of the right upper lobe infiltrate but new‐onset bilateral pleural effusions (Figure 2a,b). Thoracentesis was performed on the right (May 8) and left (May 10). Both effusions were bloody and exudative; however, the right was neutrophil‐dominant, whereas the left was lymphocyte‐dominant (Table 2). Cytological examination of both effusions was negative for malignancy. Due to the bloody effusions, only edoxaban was discontinued, and haemostatic agents were administered. Aspirin was continued following consultation with the cardiovascular surgery team. A follow‐up chest CT on May 21 demonstrated partial resolution of the pleural effusions (Figure 2c,d). Given the radiological improvement and resolution of fever and hypoxaemia, the patient was discharged on May 23.

**TABLE 2:** Characteristics of bilateral pleural effusions.

Parameter	Right (May 8)	Left (May 10)	Unit
Macroscopic appearance	Bloody	Bloody	
Biochemistry			
Total protein	4.1	3.8	g/dL
LDH	320	180	IU/L
Glucose	98	102	mg/dL
Adenosine deaminase	25	22	U/L
CEA	1.2	1	ng/mL
Cell count			
Nucleated cells	8500	2100	/μL
Red blood cells	0.02	0.05	×10^6^/μL
Haematocrit	0.18	0.44	%
Cell differentiation			
Neutrophils	85	10	%
Lymphocytes	10	90	%
Monocytes/others	5	0	%
Microbiology			
Gram stain	No organisms	No organisms	
Culture	No growth	No growth	
Acid‐fast bacilli	Negative	Negative	
Pathology			
Cytology	Negative	Negative	

Abbreviations: CEA, carcinoembryonic antigen; LDH, lactate dehydrogenase.

The patient remained stable as an outpatient for 3 months. By August 29, the left effusion had worsened and concurrent chest CT revealed a small amount of pericardial effusion (Figure 2e,f). Pericardiocentesis was not performed due to the small fluid volume; therefore, the character of the pericardial fluid could not be assessed. A repeat left thoracentesis yielded bloody, lymphocyte‐dominant fluid, consistent with prior findings. Echocardiography demonstrated preserved left ventricular function. CRP, which had normalised after discharge, had risen to 0.48 mg/dL (institutional upper limit of normal, 0.30 mg/dL). The 2015 European Society of Cardiology guidelines require at least two of five criteria for a diagnosis of PCIS [6]; our patient fulfilled two: pericardial effusion on CT and pleural effusion with an elevated CRP. Fever, pericarditic or pleuritic chest pain and pericardial or pleural rubs were absent.

Late‐onset Dressler syndrome was suspected, and diagnostic therapy with ibuprofen (600 mg/day) was initiated. Follow‐up imaging confirmed significant improvement (Figure 2g,h).

## 3 Discussion

In pneumococcal pneumonia, pulmonary parenchymal inflammation extends to the pleura, leading to a protein‐rich, neutrophil‐dominant parapneumonic effusion [2]. Conversely, Dressler syndrome is driven by immunological reactions, typically presenting as lymphocyte‐dominant exudates [3]. In the present case, while the pneumonia showed radiological improvement with antibiotic therapy, the severe initial inflammation likely led to the delayed manifestation of pleurisy, resulting in the subsequent development of pleural effusions.

Though both effusions appeared grossly haemorrhagic, their red blood cell (RBC) counts were relatively modest (0.02 × 10^6^/μL on the right and 0.05 × 10^6^/μL on the left), yielding calculated pleural fluid haematocrits of 0.18% and 0.44%, respectively. As these are far below the diagnostic threshold for haemothorax, it is possible that the patient's antithrombotic‐related bleeding tendency exacerbated microvascular oozing within the inflamed pleura, thereby imparting a bloody appearance to these exudative effusions.

According to the 2015 European Society of Cardiology guidelines, PCIS, which encompasses Dressler syndrome, typically develops after a latent period of a few weeks to months [6]. The pathophysiology of this syndrome is widely considered to be immune‐mediated: cardiac injury, such as CABG, leads to the release of sequestered cardiac antigens, which triggers the formation of anti‐heart autoantibodies [7, 8]. The time required for this immunological sensitization inherently creates a prolonged latent period between the initial injury and the onset of clinical symptoms [8].

In our case, the onset occurred approximately 12 months after CABG, which exceeds not only the usual latent period but also the 9‐month interval described in a previous report of PCIS following thymectomy [9]. We hypothesise that the patient developed long‐standing latent immune sensitisation following his CABG. The intense systemic inflammatory response induced by the severe pneumococcal pneumonia likely acted as a necessary catalyst, reactivating this dormant autoreactive process and culminating in the delayed manifestation of Dressler syndrome.

The left‐sided predominance of the effusion in our case is consistent with the reported distribution of PCIS‐related effusions, which are unilateral in two thirds of cases and left‐sided in the large majority; notably, in that series no patient with Dressler syndrome had a right‐sided unilateral effusion, although 17% of the remaining PCIS cases did [10]. The exudative and lymphocyte‐predominant character of the left effusion likewise matches the reported profile, in which nearly all such effusions are exudates and three quarters are lymphocyte‐predominant [10].

When encountering bilateral bloody pleural effusions, clinicians must navigate a broad spectrum of differential diagnoses, including malignant dissemination, pulmonary embolism, trauma and tuberculous pleurisy (pleural ADA was not elevated). In the present case, the definitive clue did not rely on the gross haemorrhagic appearance, but rather on the rare and striking finding of discordant pleural fluid cellular profiles between the two hemithoraces: a neutrophil‐dominant profile on the right and a lymphocyte‐dominant profile on the left. This distinct cellular disparity strongly argued against a single, uniform systemic process—such as carcinomatosis or a massive pulmonary embolism—and instead demonstrated that two independent, localised thoracic pathologies were occurring simultaneously.

In this case, the selective use of ibuprofen monotherapy (600 mg/day, which is the maximum dose covered by Japanese medical insurance) led to a significant improvement in the left pleural and pericardial effusions, with no recurrence during 10 months of follow‐up after the initiation of ibuprofen.

While this favourable clinical response suggests an immune‐mediated inflammatory process, we acknowledge the diagnostic uncertainty regarding the precise aetiology, since a response to non‐steroidal anti‐inflammatory drugs is common in pleuropericarditis of any cause [6]. A reactive pleuropericarditis following the preceding pneumonia (post‐infectious serositis) is a plausible alternative, as autoimmune pleuropericarditis has been reported after community‐acquired pneumonia [11].

Nevertheless, given the patient's prior CABG, we consider late‐onset Dressler syndrome to be the most likely clinical diagnosis rather than a definitive one. The two mechanisms are not mutually exclusive: An immune‐mediated predisposition related to the prior cardiac injury may have been unmasked by the systemic inflammation accompanying the contralateral pneumonia. While the lymphocyte‐predominant profile and the prompt response to ibuprofen support an immune‐mediated process, these findings do not allow us to attribute it definitively to the prior cardiac surgery rather than to the preceding infection. It should also be noted that no pericardial effusion was present at the time of the initial thoracenteses, and that the left effusion was attributed to a post‐cardiac injury process only retrospectively, once the recurrence in August fulfilled the PCIS criteria.

This case offers a crucial clinical lesson for managing bilateral pleural effusions. There is a frequent clinical pitfall to assume that bilateral effusions always stem from a single systemic aetiology, such as heart failure, renal failure or malignancy. However, as demonstrated here, bilateral effusions can arise from entirely different, independent localised pathologies that require separate therapeutic strategies—specifically, targeted antibiotic therapy for the right side and anti‐inflammatory treatment for the left. Therefore, even when bilateral effusions present with an identical gross appearance (such as being bilaterally bloody), a bilateral thoracentesis and detailed cellular analysis are highly recommended if the clinical or radiological presentation is atypical.

## Author Contributions

Y.N. managed the patient and drafted the manuscript. S.H., M.Y. and Y.A. contributed to the clinical analysis and critically reviewed the manuscript for important intellectual content. All authors have read and approved the final manuscript.

## Funding

The authors have nothing to report.

## Consent

The authors declare that written informed consent was obtained for the publication of this manuscript and accompanying images using the consent form provided by the journal.

## Conflicts of Interest

The authors declare no conflicts of interest.

## Data Availability

The data that support the findings of this case report are not publicly available in order to protect the privacy of the patient. Anonymised data are available from the corresponding author on reasonable request.
